# The use of phages for the biosynthesis of metal nanoparticles and their biological applications: A review

**DOI:** 10.1111/jcmm.18383

**Published:** 2024-06-04

**Authors:** Seyed Soheil Hosseininasab, Mahin Naderifar, Majid Reza Akbarizadeh, Mohammadjavad Rahimzadeh, Simin Soltaninejad, Zohre Makarem, Naghmeh Satarzadeh, Amin Sadeghi Dousari

**Affiliations:** ^1^ Faculty of Pharmacy Kerman University of Medical Sciences Kerman Iran; ^2^ School of Nursing & Midwifery Zabol University of Medical Sciences Zabol Iran; ^3^ School of Medicine Zabol University of Medical Sciences Zabol Iran; ^4^ School of Nursing & Midwifery Bam University of Medical Sciences Bam Iran; ^5^ School of Medicine Kerman University of Medical Sciences Kerman Iran; ^6^ Noncommunicable Diseases Research Center Bam University of Medical Sciences Bam Iran; ^7^ Department of Pharmaceutical Biotechnology, Faculty of Pharmacy Kerman University of Medical Sciences Kerman Iran; ^8^ Medical Mycology and Bacteriology Research Center Kerman University of Medical Sciences Kerman Iran

**Keywords:** biological applications, biosynthesis, metal nanoparticles, phage

## Abstract

Nowadays, the use of biological methods of synthesis of nanoparticles as substitutes for methods that use high energy and consumption of expensive and dangerous materials is of interest to researchers all over the world. Biological methods of synthesising metal nanoparticles are very important because they are easy, affordable, safe, environmentally friendly and able to control the size and shape of nanoparticles. One of the methods that is of interest today is the use of bacteriophages as the most abundant organisms in nature in the synthesis of metal nanoparticles. Nanomaterials biosynthesized from phages have shown various clinical applications, including antimicrobial activities, biomedical sensors, drug and gene delivery systems, cancer treatment and tissue regeneration. Therefore, the purpose of this review is to investigate the biosynthesis of metal nanoparticles with phages and their biomedical applications.

## 1 INTRODUCTION

In the past decades, because the science of nanoparticles is a link between atomic or molecular structures and materials in bulk, it has generated a lot of scientific interest among researchers around the world. Nanoparticles have various applications due to their unique properties such as increased reactivity, increased surface area relative to volume and high sensitivity.^1^ Nanomaterials are used in various fields such as agriculture, environment, biotechnology, pharmaceuticals and medicine.^2^ Nanoparticles have many activities, including antimicrobial, anti‐inflammatory, anti‐tumour, antioxidant, drug manufacturing, etc.^3^, ^4^, ^5^ Nanoparticles are particles smaller than 100 nm that are synthesized in different ways.^6^

Among the nanomaterials, metal nanoparticles have received considerable attention from researchers due to properties such as resonant electron oscillation and optical properties. This type of nanoparticle, the ability to integrate into the biological system with non‐toxicity, has made a significant impact on biological and pharmaceutical research.^7^ Various metal nanoparticles such as silver, gold, copper, iron, cobalt, bismuth, etc. have been synthesized using various methods and sources and have shown unique applications in various fields, especially medical and pharmaceutical science.^8^

Nanoparticle synthesis methods are very important because to obtain the maximum amount of nanoparticles, morphology and small size of nanoparticles, various factors such as temperature, concentration of supernatant, extract concentration, stirring time and pH have an effect on the synthesis of nanoparticles. There are several methods of nanoparticle synthesis including biological, chemical and physical.^9^, ^10^, ^11^, ^12^ From chemical and physical methods using heavy reducing agents such as hydrazine and sodium borohydride, organic deactivators such as thiophenol and thiourea, which have destructive effects on the environment if synthesized on a large scale. Therefore, alternative synthesis methods that are less toxic and environmentally friendly should be used.^13^

Biological synthesis or green synthesis for the biosynthesis of metal nanoparticles is an innovation that has attracted the attention of researchers in recent years because it is a substitute for the use of dangerous chemicals. Biological synthesis methods have advantages such as easy synthesis, safe, cheap, high yield and environmentally friendly. In green or biological synthesis methods, plant extracts (including flowers, fruits, leaves, stems and roots) and microorganisms such as fungi, yeasts, microalgae, bacteria and bacteriophages are used. Bacteriophages are one of the microorganisms that have been noticed in recent years due to their high potential for the biosynthesis of metal nanoparticles.^14^

Bacteriophages or phages are viruses that infect bacterial cells and multiply in them. These viruses include the head, legs, and tails, and their genome is inside a protein shell called capsid.^15^ Phages have all the common characteristics of viruses, such as they have a relatively small genome, they use the host for their reproduction, they do not reproduce beyond their limit, and they are specific to the host cell.^16^ Phages are very diverse in terms of genomic organisation, morphology, and size, and considering that they exist everywhere in the environment, they are the most abundant biological agents on Earth.^15^ The aim of this study is to examine various nanoparticles synthesized by phages, the characteristics of nanoparticles, and their biomedical effects.

Different studies on the synthesis of various nanoparticles by phages and their biological applications have been conducted worldwide, which are described and discussed below (Table 1).

**TABLE 1:** Characteristics of different nanoparticles synthesized by phages.

Type of phage	Phage host	The origin of synthesis	Structure of nanoparticles	Analyzes performed	Size (nm)	Shape	Applications	Ref.
C3 (*Podoviridae*)	*Salmonella* serovar Paratyphi B	HAuCl_4_	Au^0^	UV–Vis, SEM, EDS, XRD, DLS	20–100	Sphere, Hexagon, Triangle, Rhomboid, Rectangular	The biosynthesized gold nanoparticles had the highest antibacterial activity against *P. aeruginosa* and the diameter of the inhibition zone was 1.4 ± 0.2 cm. The biosynthesized gold nanoparticles in concentrations of 0.1 mM and 0.2 mM inhibited 70% and 80% of biofilm in *P. aeruginosa* strains forming biofilm, respectively.	^17^
ZCSS1 (*Myoviridae*)	*Staphylococcus sciuri*	AgNO_3_	Ag^+^	TEM	10–30	Regular	The biosynthesized silver nanoparticles with MIC and MBC were 12.5 μg/mL against *S. sciuri*, and the diameter of the inhibition zone was 12.6 mm. The biosynthesized silver nanoparticles caused biofilm rupture in *S. sciuri*.	^18^
P1 and ɸ6	*Escherichia coli* (P1), *Pseudomonas syringae* (ɸ6)	FeCl_3_, FeCl_2_	Fe_3_O_4_	UV–vis, SEM, XRD	‐	‐	‐	^19^
M13	‐	Magnetosome	Fe_3_O_4_	TEM, AFM	~30	Sperm‐like, Chain‐like	‐	^20^
M13	‐	Fe^2+^, Fe^3+^	FeO	HRTEM, XRD	‐	Cubic	‐	^21^
M13	‐	CuCl_2_, Na_2_S	CuS	TEM, EDX	2–7	Cubic	‐	^22^
M13	‐	Zn(OH)_2_	ZnO	UV–vis, TEM, SEM, EDX, XRD	20–40	Cubic	‐	^23^
M13	‐	MnAc_2_	MnO_2_	TEM, XPS	20–30	‐	‐	^24^
M13	‐	Zn(NO_3_)_2_.6H_2_O	ZnO	TEM	46 ± 18–54 ± 21	Spherical	‐	^25^
M13	‐	Bi(NO_3_)_3_·5H_2_O	Bi^3+^	UV–vis, FTIR, TEM	3–7	Spherical	‐	^26^

Abbreviations: DLS, dynamic light scattering; EDS, energy‐dispersive spectroscopy; FTIR, fourier‐transform infrared spectroscopy; HRTEM, high‐resolution transmission electron microscopy; SEM, scanning electron microscopy; TEM, transmission electron microscopy; UV–Vis, ultraviolet–visible; XPS, x‐ray photoelectron spectroscopy; XRD, x‐ray diffraction analysis.

## 2 ADVANTAGES AND DISADVANTAGES OF USING PHAGES FOR THE BIOSYNTHESIS OF METAL NANOPARTICLES

Phages as new organisms with high potential for the biosynthesis of metal nanoparticles have advantages and disadvantages.^27^ The advantages of using phages as biosynthesizers of metal nanoparticles, they are organisms with great diversity and are present everywhere,^28^ they are methods that are friendly to the environment,^29^ and they have special enzymes and structures to increase the efficiency of synthesis^30^ and their special selectivity because phages are specifically attached to bacteria, which makes the biosynthesis process more accurate.^31^ The proteins or peptides in bacteriophages act as suitable capping, reducing and stabilising factors in the biosynthesis of nanoparticles, which causes bacteriophages employed as natural agents in the production of nanoparticles.^17^, ^32^, ^33^ Furthermore, the use of phages has disadvantages such as the use of phages may create limitations in the size and shape of nanoparticles, the complexity of the biosynthesis process, the need for special conditions, and limitations in the choice of metals, which in some phages only biosynthesize a specific metal.^30^ The few disadvantages involving bacteriophage mediated nanoparticle synthesis are the long synthesis time, and loss of purification nanoparticles step. Consequently, they can interact with the immune system and induce harmful immune responses, which is a concern for clinical applications of bacteriophage‐mediated nanoparticle synthesis.^17^, ^18^, ^23^, ^34^, ^35^

## 3 HOW ARE METAL NANOPARTICLES BIOSYNTHESIZED USING PHAGES?

Various plants and microorganisms are used in the biological method of nanoparticle synthesis. Microorganisms that are usually used in the biosynthesis of metal nanoparticles are bacteria and fungi.^11^ In recent years, researchers around the world have focused on the high abilities of phages and the potential of these organisms in the biosynthesis of metal nanoparticles. Generally, for the biosynthesis of metal nanoparticles by phages, the desired phages are first cultured on the culture medium containing host bacteria and incubated for 1 day and night. After the phages are grown, they are purified in steps using a centrifuge. Finally, the desired phages are cultured in the culture medium containing various ions and the phages act as a regenerator for the biosynthesis of nanoparticles. The first sign in the biosynthesis of metal nanoparticles with phages is the change in rank in the mixture of phage and desired ion, and UV–vis, TEM, SEM, EDS, XRD, and DLS analyses are used for more accurate characterisations.

## 4 CHARACTERISTICS OF NANOPARTICLES SYNTHESIZED BY PHAGES

**FIGURE 1:**
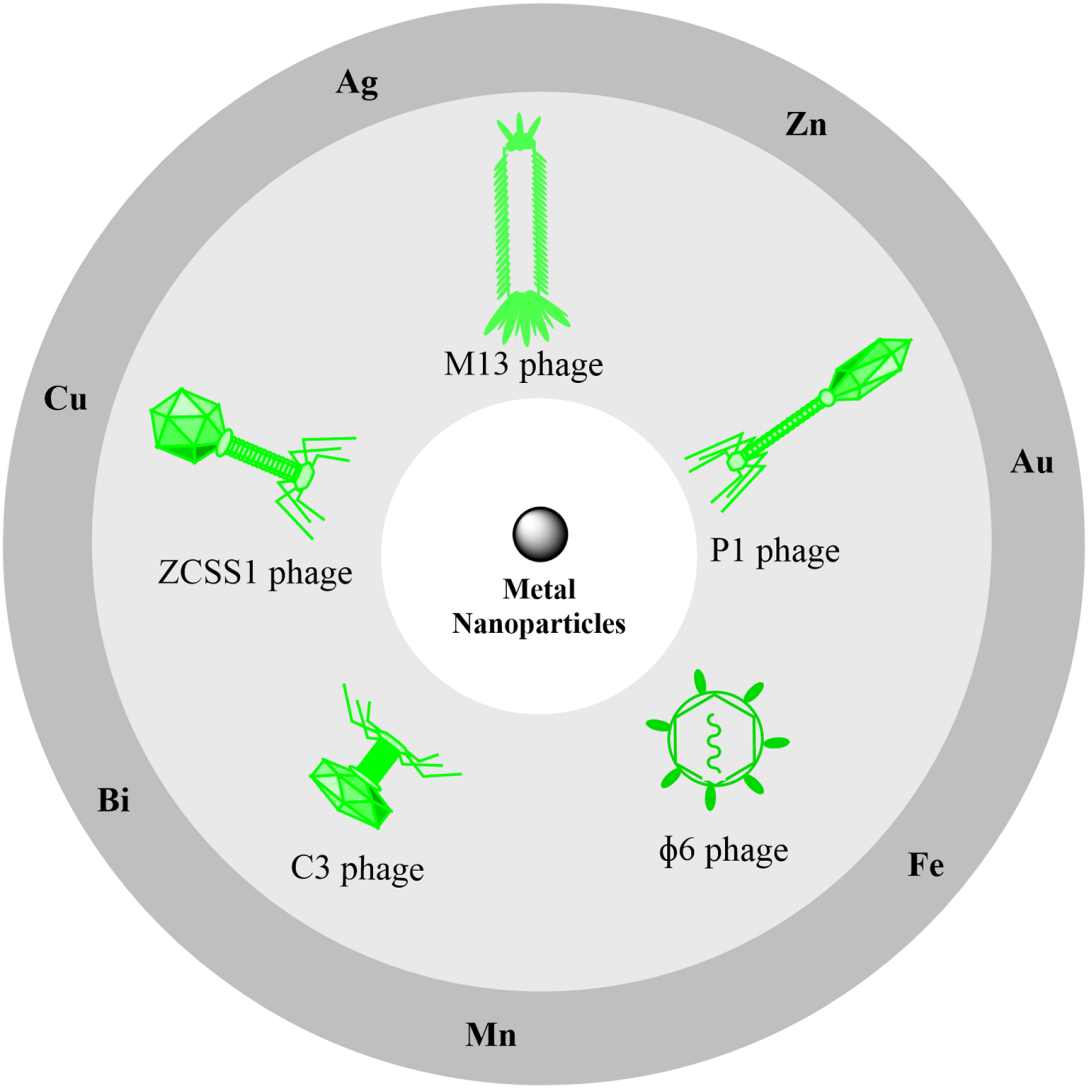
Types of phages biosynthesising metal nanoparticles.

The review of various studies revealed that different metal nanoparticles were synthesized by phages. The most used phage in the studies was phage M13, but other phages such as C3, ZCSS1, P1, and ɸ6 have also been used (Figure 1). In the studies, it has been reported that the studied phages were isolated from their hosts in Gram‐negative bacteria such as *Salmonella serovar Paratyphi B*, *Escherichia coli*, and *Pseudomonas syringae*, as well as Gram‐positive bacteria such as *Staphylococcus sciuri*. Various analyses such as FTIR, UV–Vis, DLS, SEM, TEM, EDS and XRD have been utilized to study the characterisations of nanoparticles. Using UV–vis analysis to check the spectrum of nanoparticles, FTIR to check surface functional groups, and EDS to check the composition of nanoparticles, biosynthesis of metal nanoparticles by phages including gold, copper, iron, silver, zinc, manganese and bismuth nanoparticles have been described in different studies. XRD analysis demonstrated various structures of biosynthesized nanoparticles with phages in the form of zero‐valent gold (Au^0^), monovalent silver (Ag^+^), magnetite and iron oxide (Fe^3^O^4^, FeO), copper monosulfide (CuS), zinc oxide (ZnO), manganese dioxide (MnO_2_), and trivalent bismuth (Bi^3+^). DLS and SEM analysis indicated that the size of the nanoparticles was between 3 and 100 nm, and also, in TEM analysis most shapes of the nanoparticles were cubic and spherical but there were also other shapes such as hexagon, triangle, rhomboid, rectangular, and regular. Studies have shown that various metal nanoparticles with different sizes and shapes biosynthesized by phages have shown significant biomedical effects such as antibacterial and antibiofilm, which are explained below.

## 5 PHAGE USED FOR THE BIOSYNTHESIS OF METAL NANOPARTICLES

Different studies have shown that different phages such as M13, C3 ZCSS1, P1 and ɸ6 have been used for the biosynthesis of different metal nanoparticles. Phage M13 has shown the ability to biosynthesize nanoparticles of iron, copper, zinc, manganese and bismuth with sizes between 2 and 54 nm and cubic shapes for iron, copper and zinc and spherical shapes for zinc and bismuth. Phage C3 has shown a high ability in the biosynthesis of gold nanoparticles with sizes of 20–100 and sphere, hexagon, triangle, rhomboid, and rectangular shapes. Phage ZCSS1 showed the ability to biosynthesize silver nanoparticles with sizes 10–40 and regular shapes. Also, phages P1 and ɸ6 showed the ability to biosynthesize iron nanoparticles, which will be explained below.

### 5.1 M13 phage

M13 is a filamentous phage that was first isolated from *Escherichia coli* in 1963. Phage M13 is a lysogenic phage belonging to the *Inoviridae* family.^36^ This phage has a length of about 900 nm and a width of about 6 nm. It has a single‐stranded DNA genome that has 9 genes that encode 11 proteins. Among 11 proteins, two proteins pVIII and pIII are the most common proteins involved in the synthesis of nanoparticles.^37^

In a study, Olszewska et al.^20^ biosynthesized Magnetite nanoparticles by phage M13. In this study, first, the culture medium containing *E. coli* K12 ER2738 bacteria was used for the propagation of phages and they were incubated overnight. Then, the phages are purified by centrifugation for 15 min at 12,000 × ***g***. In the next step, phages were cultured in the vicinity of magnetosomes isolated from *M. gryphiswaldense* MSR‐1 strain and *M. magneticum* AMB‐1 strain. The results showed that phages biosynthesized Magnetite (Fe_3_O_4_) nanoparticles using pIII protein. In another study, Rawlings et al.^21^ biosynthesized iron oxide nanoparticles using phage M13. In this study, phage M13 was cultured in a medium containing *E. coli* bacterium. The results showed that M13 phage biosynthesized iron oxide magnetic nanoparticles through pIII protein and adhirons. Moon et al.^25^ biosynthesized zinc oxide nanoparticles using M13 phage. In this study, phages were placed in the vicinity of zinc hydroxide solution at a concentration of 1 × 10^11^ pfu/μL. The results of this study showed that phage M13 biosynthesized zinc oxide nanoparticles using pIII protein. Using M13 phage, copper sulphide nanoparticles were biosynthesized by Zaman et al.^22^ Phage M13 was incubated at a concentration of 2.5 × 1011 pfu/μL in the medium containing copper chloride and sodium sulphide. The results showed that copper sulphide nanoparticles were biosynthesized on pVIII protein. In a study, Żelechowska et al.^23^ biosynthesized zinc oxide nanoparticles using M13 phage. In this study, phage M13 was reproduced in the presence of *E. coli* ER2738 and then incubated with zinc hydroxide. The result demonstrated that phage M13 has biosynthesized zinc oxide nanoparticles using pVIII and pIII proteins. The synthesis of manganese dioxide nanoparticles was also biosynthesized using M13 phage. Phage M13 was incubated overnight in the presence of *E. coli* TG‐1 and duplicated. Then the purified phages were mixed with manganese acetate solution and filtered for 6 to 8 h after mixing. The results displayed that manganese dioxide nanoparticles were biosynthesized on the pVIII protein.^24^ Furthermore, in another study, Vera‐Robles et al.^26^ biosynthesized bismuth nanoparticles using phage M13. In this study, phages were reproduced in an LB medium containing *E. coli* strain. Then 15 microliters of phage M13 at a concentration of 1 × 10^12^ pfu were incubated overnight in the vicinity of 15 microliters of bismuth nitrate. The results of this study exhibited that bismuth nanoparticles were biosynthesized on pVIII protein in phage M13.

### 5.2 C3 phage

In the *Caudovirales* order, there are most phages that have two and contain double‐stranded DNA. This order is divided into three families, including *Myoviridae* phages with complex contractile tails, *Siphoviridae* phages with flexible and long tails, and *Podoviridae* with short or no tails. The phages with C3 morphology belong to the *Podoviridae* family, which have an elongated chain of about 90 to 223 nm and a short tail. This type of phages infects gram‐negative intestinal bacteria and gram‐positive lactococci.^38^

In a study, Ahiwale et al.^17^ biosynthesized gold nanoparticles using phage C3. First, the phages were cultured in the medium containing *Salmonella* serovar Paratyphi B and incubated at 37 C overnight. 5 mL of the purified phages were mixed at a concentration of 6 × 10^11^ PFU/ml in 2 mL of medium containing chloroauric acid solution and incubated for 74 h. The results displayed that phage C3 has biosynthesized gold nanoparticles by changing the colour from yellow to red in chloride solution.

### 5.3 ZCSS1 phage

ZCSS1 belongs to the *Myoviridae* family and is a temperate phage with lytic activity. The host of this phage is *Staphylococcus sciuri*. ZCSS1 phage has high stability against harsh chemical and physical conditions.^39^

Abdelsattar et al.^18^ biosynthesized silver nanoparticles using phage ZCSS1 isolated from *S. sciuri*. In this study, 20 mL of silver nitrate (1 mM) was mixed with 200 μL of phage ZCSS1 at a concentration of 10^10^ PFU/mL, then the resulting mixture was kept at room temperature for 5 h in the presence of light at 300 rpm. The results displayed that silver nanoparticles were biosynthesized using phage ZCSS1.

### 5.4 P1 and ɸ6 phages

P1 is a temperate tailed phage that belongs to the *Myoviridae* family and has a genome of 93.6 kb. P1 is one of the types of lytic phases that infect different species of *Enterobacteriaceae* and *Rhizobiaceae* family.^40^ Phage Φ6 is a lytic phage belonging to the *Cystoviridae* family, which has a double‐stranded RNA genome with a length of 13.5 kb. This phage infects *Pseudomonas syringae* and is one of the prominent features of the lipid membrane around its capsid.^41^

Działak et al.^19^ biosynthesized iron oxide magnetic nanoparticles using P1 and ɸ6 phages isolated from *E. coli* (P1), *Pseudomonas syringae* (ɸ6). In this study, 100 mL of iron chloride was mixed with 1 mL of phage suspension at a concentration of 1 × 10^10^ PFU, then it was stirred at a temperature of 40 C with 600 rpm to cause a colour change, and finally, magnetic nanoparticles were separated using a magnet. The results displayed that magnetic iron nanoparticles were biosynthesized using P1 and ɸ6 phases.

## 6 BIOMEDICAL APPLICATIONS OF METAL NANOPARTICLES SYNTHESIZED BY PHAGES

**FIGURE 2:**
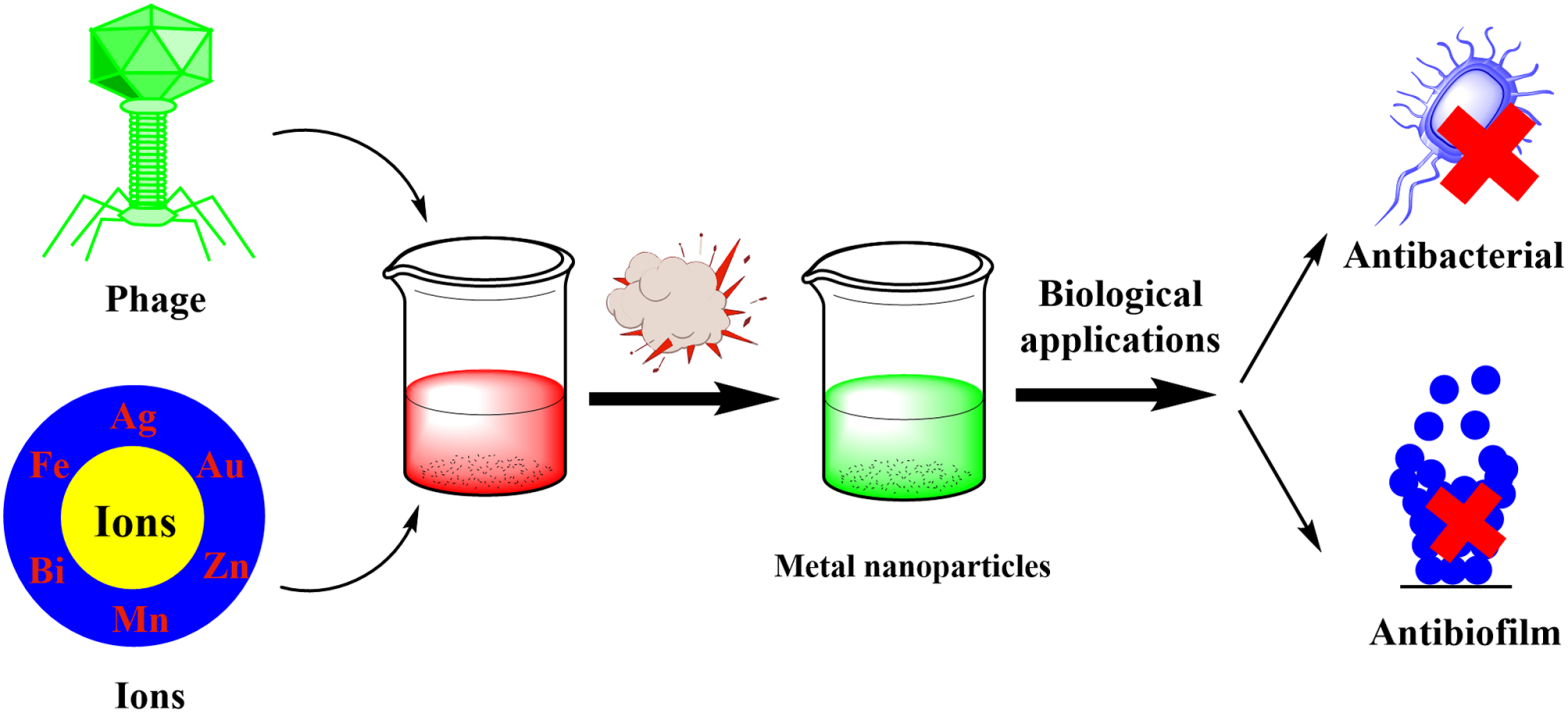
Schematic image of the synthesis of different nanoparticles synthesized by phages.

In the reviewed studies of the biomedical applications of metal nanoparticles synthesized with phages, their antibacterial and antibiofilm activity can be mentioned (Figure 2).

### 6.1 Antibacterial effects

The antibacterial activity of gold nanoparticles biosynthesized with phage C3 was examined by Ahiwale et al.^17^ on the bacteria *Pseudomonas aeruginosa*, *Klebsiella pneumoniae*, S. *Serovar paratyphi* B, *Proteus vulgaris*, and *E*. *coli* by well agar diffusion method. In this study, wells were created in a nutrient agar medium, then the desired bacteria were cultured. Gold nanoparticles biosynthesized with phage C3 were added to the wells in concentrations of 50 μL and 100 μL and incubated at 37°C for 24 h, and then the diameter of the growth inhibition zone was measured. The results demonstrated that the biosynthesized gold nanoparticles had the highest antibacterial activity against *P. aeruginosa* and the diameter of the inhibition zone was 1.4 ± 0.2 cm.

In another study, Abdelsattar et al.^18^ investigated the antibacterial activity of silver nanoparticles biosynthesized with ZCSS1 phage against *Staphylococcus sciuri* by serial dilution method. In this study, the target bacterium was cultured in a broth medium, and by adding 20 μL biosynthesized silver nanoparticles, the minimum inhibitory concentration (MIC) and minimum bactericidal concentration (MBC) were investigated. Also, the target bacterium was cultured on the medium, and by creating a well in the medium, 20 mL of silver nanoparticles with concentrations of 50% and 100% were added and incubated. The results indicated that the biosynthesized silver nanoparticles with MIC and MBC were 12.5 μg/mL against *Staphylococcus sciuri*, and the diameter of the inhibition zone was 12.6 mm.

### 6.2 Antibiofilm effects

Biofilm refers to a state in which bacterial cells are attached to living and non‐living surfaces and surrounded by a matrix of extracellular materials. By forming biofilm, bacteria become 10 to 1000 times more resistant to antimicrobial substances and antibiotics. It has been reported that 80% of bacterial infections in infected people are related to biofilm‐forming bacteria.^42^ *P. aeruginosa*, *E. coli*, *Staphylococcus epidermidis*, *Proteus mirabilis*, *Enterococcus faecalis*, *K. pneumonia*, *Staphylococcus aureus* and *Streptococcus viridans* can be mentioned among the most important bacteria that cause biofilm.^43^ Nowadays, due to the formation of biofilm in bacteria and the ineffectiveness of antibiotics, the synthesis of metal nanoparticles with antibiofilm properties has been the focus of researchers.^42^

Antibiofilm activity of biosynthesized gold nanoparticles with C3 phage was investigated on *P. aeruginosa* bacteria forming biofilm by microplates method. First, the desired bacteria were cultured in nutrient broth medium in 96‐well microplates, and then gold nanoparticles biosynthesized with C3 phage were added in concentrations of 0.05, 0.1 and 0.2 mM and incubated at 37°C for 24 h, and finally after incubations were stained with 0.01% acridine orange stain. The results revealed that biosynthesized gold nanoparticles in concentrations of 0.1 mM and 0.2 mM inhibited 70% and 80% of biofilm in *P. aeruginosa* strains forming biofilm, respectively.^17^

The antibiofilm activity of silver nanoparticles biosynthesized with ZCSS1 phage against *Staphylococcus sciuri*. In this study, the target bacterium was cultured in a sterile plastic cover medium and was added 6.2 μg/mL biosynthesized silver nanoparticles and investigated with a scanning electron microscope (SEM). The results of SEM imaging demonstrated that biosynthesized silver nanoparticles caused biofilm rupture in *S. sciuri*.^18^

## 7 CONCLUSION AND PERSPECTIVES

Nowadays, the synthesis of metal nanoparticles using biological methods and investigating their biomedical applications has attracted the attention of various researchers around the world. One of the resources that has been considered for the biosynthesis of metal nanoparticles in recent years is the use of phages as the most abundant organism in nature. The review of various studies showed that various metal nanoparticles from the journal gold, copper, iron, silver, zinc, manganese and bismuth were biosynthesized by phages. The most phage used in the studies was phage M13, but other phages such as C3, ZCSS1, P1, and ɸ6 were also used. In the M13 phase, pVIII and pIII proteins showed a significant role in the reduction of metal nanoparticles. The remarkable thing about the nanoparticles biosynthesized with phages was the size of the nanoparticles, which was between 3 and 100 nm. Most of the shapes of nanoparticles were cubic and spherical, but there were also other shapes such as hexagonal, triangular, rhombic, rectangular, and regular. Metal nanoparticles biosynthesized with phages showed significant antibacterial and antibiofilm activity. It seems that the use of phages for the biosynthesis of metal nanoparticles is a biological method with high potential that should be considered. According to the results of the studies, the M13 phage has a higher potential for the biosynthesis of different nanoparticles as a green synthesis method among all phages. Also, it seems that nanoparticles biosynthesized from phages have a significant antibacterial effect on *S. sciuri* and *P. aeruginosa*, as well as an antibiofilm effects on *S. aureus*, *S. epidermidis*, *S. sciuri*, *E. faecalis* and *S. viridans* as Gram‐positive bacteria and *P. aeruginosa*, *E. coli*, *P. mirabilis*, *K. pneumonia*, and *P. aeruginosa* as gram‐negative bacteria. The use of biosynthesis of various nanoparticles using microorganisms as a green synthesis method is growing rapidly in recent years because they are easy to synthesize, easy to maintain, low cost, non‐toxic and safe and environmentally friendly production. However, due to the study of phages is a new method for the biosynthesis of nanoparticles, more studies are needed. Although several studies have presented the potential of using microorganisms as agents for synthesising nanoparticles and their use in medicine and various treatments, the use of phages as a new organism in studies requires various investigations such as biocompatibility, toxicity, effects on the immune system, and different organs of the body.

## AUTHOR CONTRIBUTIONS

**Seyed Soheil Hosseininasab:** Writing – original draft (equal); writing – review and editing (equal). **Mahin Naderifar:** Writing – review and editing (equal). **Majid Reza Akbarizadeh:** Writing – review and editing (equal). **Mohammadjavad Rahimzadeh:** Writing – review and editing (equal). **Simin Soltaninejad:** Writing – review and editing (equal). **Zohre Makarem:** Writing – review and editing (equal). **Naghmeh Satarzadeh:** Writing – review and editing (equal). **Amin Sadeghi Dousari:** Data curation (equal); investigation (equal); supervision (equal); writing – original draft (equal); writing – review and editing (equal).

## FUNDING INFORMATION

This work has no funding.

## CONFLICT OF INTEREST STATEMENT

The author has no conflict of interest to declare.

## Data Availability

All data have been included within this manuscript.
